# Clinical and radiological predictors of epidermal growth factor receptor mutation in nonsmall cell lung cancer

**DOI:** 10.1002/acm2.13107

**Published:** 2020-12-12

**Authors:** Yutao Dang, Ruotian Wang, Kun Qian, Jie Lu, Haixiang Zhang, Yi Zhang

**Affiliations:** ^1^ Department of Thoracic Surgery Xuanwu Hospital Capital Medical University Beijing China; ^2^ Department of Thoracic Surgery Shijingshan Hospital of Beijing City Shijingshan Teaching Hospital of Capital Medical University Beijing China; ^3^ Department of Radiology Xuanwu Hospital Capital Medical University Beijing China; ^4^ Center for Applied Mathematics Tianjin University Tianjin China

**Keywords:** epidermal growth factor receptor mutation, nomogram, non‐small‐cell lung cancer, prediction model, radiomics

## Abstract

**Purpose:**

To determine the prognostic factors of epidermal growth factor receptor (EGFR) mutation status in a group of patients with nonsmall cell lung cancer (NSCLC) by analyzing their clinical and radiological features.

**Materials and methods:**

Patients with NSCLC who underwent EGFR mutation detection between 2014 and 2017 were included. Clinical features and general imaging features were collected, and radiomic features were extracted from CT data by 3D Slicer software. Prognostic factors of EGFR mutation status were selected by least absolute shrinkage and selection operator (LASSO) logistic regression analysis, and receiver operating characteristic (ROC) curves were drawn for each prediction model of EGFR mutation.

**Results:**

A total of 118 patients were enrolled in this study. The smoking index (*P* = 0.028), pleural retraction (*P* = 0.041), and three radiomic features were significantly associated with EGFR mutation status. The areas under the ROC curve (AUCs) for prediction models of clinical features, general imaging features, and radiomic features were 0.284, 0.703, and 0.815, respectively, and the AUC for the combined prediction model of the three models was 0.894. Finally, a nomogram was established for individualized EGFR mutation prediction.

**Conclusions:**

The combination of radiomic features with clinical features and general imaging features can enable discrimination of EGFR mutation status better than the use of any group of features alone. Our study may help develop a noninvasive biomarker to identify EGFR mutation status by using a combination of the three group features.

## 1 INTRODUCTION

Lung cancer is the leading cause of cancer‐related death worldwide. Approximately 70% of lung cancer patients were diagnosed after clinical symptoms caused by local advanced stage or metastasis. The 5‐year survival rate of these patients is only approximately 16%.^1^, ^2^ With the development of targeted therapy, the survival time and quality of life of some lung cancer patients have greatly improved. Targeted therapy relies on gene detection, and at present, most of the tissues used for gene detection are specimens obtained by surgical excision or biopsy. For some patients, biopsy specimens may be the only tissue specimens that can be used for gene detection, but because of the small or low DNA content of tissue samples, it may be impossible to carry out gene detection, or incorrect detection results may be obtained.^3^ Furthermore, due to tumor heterogeneity, there may be a positive mutation in the EGFR gene that is negative at the tissue biopsy site.^4^, ^5^, ^6^ Although some clinical studies have suggested that adenocarcinoma, nonsmoking status, female sex, and Asian race are predictors of EGFR mutations,^7^, ^8^, ^9^ studies have also shown that adenomatous hyperplasia, atypical adenomatous hyperplasia, adenocarcinoma in situ, and squamous dominant adenocarcinoma frequently carry EGFR mutations.^10^, ^11^, ^12^, ^13^, ^14^, ^15^ These results provide a reference for predicting the mutation status of lung cancer genes, but powerful noninvasive predictive markers are still lacking. Radiomics refers to the extraction of sub‐visual yet quantitative image features with the intent of creating mineable databases from radiological images.^16^ Some features have even been shown to identify genomic alterations within tumor DNA, a field that is now called “radiogenomics”.^17^ These features can identify specific driving mutations and changes in biological pathways. Recently, radiomic features extracted from chest CT have been used to predict EGFR mutation in NSCLC in some studies,^18^, ^19^, ^20^, ^21^ but most of these studies included only a few radiomic features in their analyses.^19^, ^20^, ^21^ Additionally, in these studies,^18^, ^19^, ^20^ only some clinical features were incorporated to improve the prediction ability of the EGFR mutation prediction model, and general imaging features were excluded. Therefore, in this study, we aimed to use reasonable statistical methods to screen meaningful features from numerous radiomic features and to establish a prediction model of EGFR mutation combined with general imaging features and clinical features.

## 2 PATIENTS AND METHODS

### 2.1 Patient selection

A total of 1292 cases of NSCLC were collected from January 2014 to December 2017. The inclusion criteria were as follows: (1) patients with detailed clinical data, including gender, age, smoking index (number of cigarettes per day * number of years of smoking), family history of lung cancer, pathological type and pathological stage (classified according to the TNM classification system of the American Join Committee on Cancer); (2) patients with a clear mutation in the EGFR gene (using the Amplification Refractory Mutation System (ARMS)), and the tissue used for mutation detection was obtained from surgical excision specimens; and (3) standard unenhanced chest CT data were obtained within 2 months before the operation, and CT was performed by the same machine under the same scanning conditions. The exclusion criteria were as follows: (1) chemotherapy or radiotherapy performed before the detection of EGFR gene mutation; (2) CT images that did not show clearly defined boundaries for pulmonary masses or pulmonary masses with atelectasis or pleural effusion; (3) the presence of EGFR gene mutations combined with other gene mutations, deletions, or rearrangements; and (4) pathological results and gene mutation status obtained from extrapulmonary metastases.

### 2.2 Chest CT examination and general imaging feature acquisition

All preoperative chest CT images were nonenhanced and acquired by one machine (Sensation Cardiac 64, Siemens Medical Solutions, Forchheim, Germany). All CT examinations were performed with the following parameters: 120 kVp; pitch, 1.2; 100–200 mAs; a 512 × 512 matrix, a B30f reconstruction kernel, 5‐mm reconstruction increments, and section thicknesses of 5 mm; voxel sizes ranged from 0.54 to 0.79 mm in the X and Y directions. Two radiologists with more than 5 years of experience blinded to the EGFR mutation status interpreted all CT images. The following characteristics should be identified: ground glass opacity (GGO), lobulation, spiculation, pleural retraction, and the air bronchogram sign. If the two radiologists disagreed, the final decision was made after analysis by another senior radiologist.

### 2.3 CT texture analysis

#### 2.3.1 Radiomic feature extraction

CT data in DICOM format were imported into 3D‐slicer software (Version 4.6.2; Surgical Planning Laboratory, Brigham and Women's Hospital, MA, USA; http://www.slicer.org). The volume of interest (VOI) was obtained by semiautomatic segmentation using the Segment Editor package. The VOI was then normalized by the package “NormalizeImageFilter.” Before feature extraction by the radiomic package (version 2.1.0), gray‐level discretization and voxel resampling were performed. All features were calculated with a fixed bin width of 25 Hounsfield Units (HU), and resampling to a voxel size of 0.6*0.6*5.0 mm^3^ was applied. The characteristics can be divided into two groups: original features: (1) shape‐based (14 features), (2) gray‐level dependence matrix (14 features), (3) first‐order statistics (18 features), (4) gray‐level co‐occurrence matrix (24 features), (5) gray‐level run‐length matrix (16 features), (6) gray‐level size zone matrix (16 features), and (7) neighboring gray tone difference matrix (5 features). Wavelet features: Features are calculated from the intensity and texture features of the original image using a wavelet filter. Therefore, the features are concentrated in different frequency ranges within the tumor volume.

#### 2.3.2 Stable radiomic feature selection

To obtain stable radiomic features, each image data point is subjected to VOI segmentation and radiomic feature extraction twice, the intraclass correlation coefficient (ICC) for each radiomic feature is calculated, and ICC > 0.75 is the stable feature.

### 2.4 Selection of prediction factors and establishment of prediction model

Patients enrolled in our study were divided into a training cohort and a validation cohort. To develop a better prediction model, we used more suitable statistical methods for predictor selection. In terms of the clinical and general imaging features, we applied a backward step‐down selection process in a logistic regression analysis to select independent prediction factors. In the radiomics model, we used minimax concave penalty (MCP)‐penalized LASSO regression analysis and tenfold cross‐validation to select predictors, and before this process the radiomic features normalization were carried out through scale function in R software (version 3.5.2, http://www.R‐project.org). A previous study showed that for statistical analysis of high‐dimensional data, MCP‐penalized LASSO regression analysis can avoid overfitting in the prediction and identify relevant variables for subsequent applications.^22^ During the process of predictor selection for the combined prediction model, to address the multicollinearity problem that may exist among the groups of data, we did not cluster or combine the radiomic features, as in previous studies.^18^, ^23^ After features normalization we performed MCP‐penalized LASSO regression analysis on all factors and ultimately obtained independent predictors. All predictors were used to develop prediction models. ROC curves were plotted, and AUC values represented the predictive ability of the models. Finally, all meaningful predictors were used to build a combined prediction model, which was compared with the radiomic feature prediction model, clinical feature prediction model, and general image feature prediction model. We also used the validation cohort to validate the discrimination ability of the prediction models.

### 2.5 Statistical analysis

Statistical analysis was performed using SPSS version 22.0 software (SPSS, Inc., IBM Company, Chicago, Illinois, USA) and R software. The means of continuous variables were compared using the Mann–Whitney U test, and Pearson chi‐square test was used for categorical variables between the EGFR (+) group and the EGFR (‐) group by SPSS. ICC was calculated using the “psych” package in R. The “MASS” package was used for logistic regression in the clinical features group and general imaging features group. The LASSO regression analysis was performed for radiomic features and combined predictor selection by the “ncvreg” package in R. The ROC curve was built by the “pROC” package and “ggplot2” package in R. A nomogram was formulated by using the package “rms” in R, and the performance of the nomogram was measured by the concordance index (C‐index), which was calculated with the “rcorrcens” package in “Hmisc” in R. The larger C‐index represented an accurate prediction. Moreover, calibration curves were plotted for the nomogram. *P* < 0.05 was set as statistically significant. The related computerized programs with R are listed in the Appendix.

## 3 Results

### 3.1 Clinical and general imaging characteristics of the patients

**Fig. 1:**
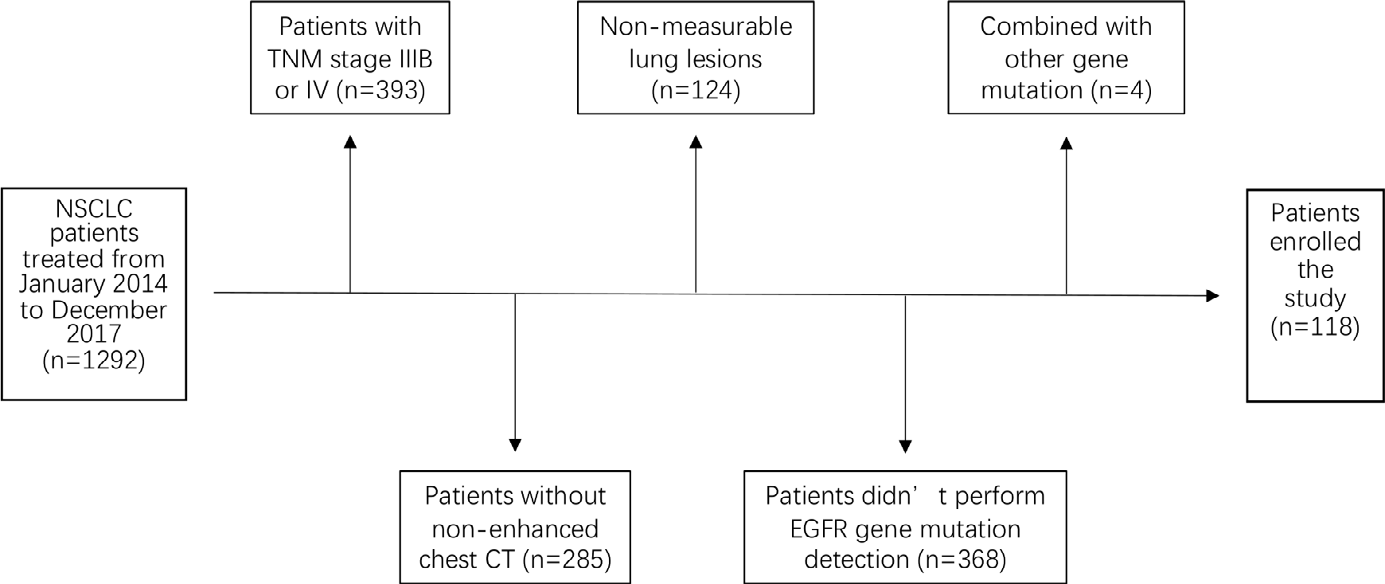
Flow chart of patient selection.

After selection, a total of 118 patients were enrolled in this study (Fig. 1). The average age of the patients was 63.82 ± 9.41. Among them, 43 (36.4%) were positive for EGFR mutation, and 75 (63.6%) were negative for EGFR mutation. There were 96 cases of adenocarcinoma (81.4%) and 22 cases of squamous cell carcinoma (18.6%). The pathological stages were as follows: stage I for 71 patients (60.2%), stage II for 21 patients (17.8%), and stage III for 26 patients (22.0%). There was no significant difference in terms of age (*P* = 0.420), family history of lung cancer (*P* = 0.139) or pathological stage (0.810) between the two groups. However, significant differences in gender (*P* = 0.022), pathological type (*P* < 0.001), and smoking index (*P* < 0.001) were found between the two groups (Table 1).

**Table 1:** Clinical features of all patients.

	EGFR (+)	EGFR (‐)	Total	*p*‐value*	OR (95%CI)
Number of patients	43	75	118		
Sex				0.022	2.426 (1.126‐5.229)
Male	17 (27.0%)	46 (73.0%)	63		
Female	26 (47.3%)	29 (52.7%)	55		
Age^#^	62.72 ± 1.54	64.45 ± 1.04		0.420	0.184 (−0.192‐0.559)
Pathological type				<0.001	0.707 (0.611‐0.818)
Adenocarcinoma	43 (44.8%)	53 (55.2%)	96		
Squamous cell carcinoma	0 (0.0%)	22 (100%)	22		
Family history				0.139	3.158 (0.716‐13.933)
Yes	5 (62.5%)	3 (37.5%)	8		
No	38 (34.5%)	72 (65.5%)	110		
Smoking index^#^	13.95 ± 8.04	381.87 ± 61.35		<0.001	1.137 (0.733‐1.538)
Stage				0.810	
IA	21 (39.6%)	32 (60.4%)	53		1.00 (reference)
IB	7 (38.9%)	11 (61.1%)	18		0.97 (0.324‐2.901)
IIA	4 (44.4%)	5 (55.6%)	9		1.219 (0.293‐5.07)
IIB	4 (33.3%)	8 (66.7%)	12		0.762 (0.203‐2.853)
IIIA	7 (26.9%)	19 (73.1%)	26		0.561 (0.201‐1.567)

EGFR, epidermal growth factor receptor; OR, odds ratio; CI, confidence interval.

^#^ Mean ± standard deviation.

* *P*‐value was based on comparison between EGFR mutation (+) group with EGFR mutation (‐) group.

As shown in Table 2, of the five general imaging features obtained from chest CT images, only pleural retraction was significantly different between the two groups (*P* = 0.003).

**Table 2:** General imaging features of all patients.

	EGFR (+)	EGFR (‐)	*P*‐value*	OR (95%CI)
Lobulation			0.627	1.209 (0.562‐2.599)
Yes	28 (60.9%)	18 (39.1%)		
No	47 (65.3%)	25 (34.7%)		
Pleural retraction			0.003	3.18 (1.458‐6.938)
Yes	26 (49.1%)	27 (50.9%)		
No	49 (75.4%)	16 (24.6%)		
GGO			0.094	2.234 (0.86‐5.808)
Yes	10 (47.6%)	11 (52.4%)		
No	65 (67.0%)	32 (33.0%)		
Air bronchogram			0.733	1.142 (0.532‐2.451)
Yes	29 (61.7%)	18 (38.3%)		
No	46 (64.8%)	25 (35.2%)		
Spiculation			0.981	0.99 (0.451‐2.176)
Yes	49 (63.6%)	28 (36.4%)		
No	26 (63.4%)	15 (36.6%)		

EGFR, epidermal growth factor receptor; GGO, ground glass opacity; OR, odds ratio; CI, confidence interval.

* *P*‐value was based on comparison between EGFR mutation (+) group with EGFR mutation (‐) group.

### 3.2 Radiomic feature selection

**Fig. 2:**
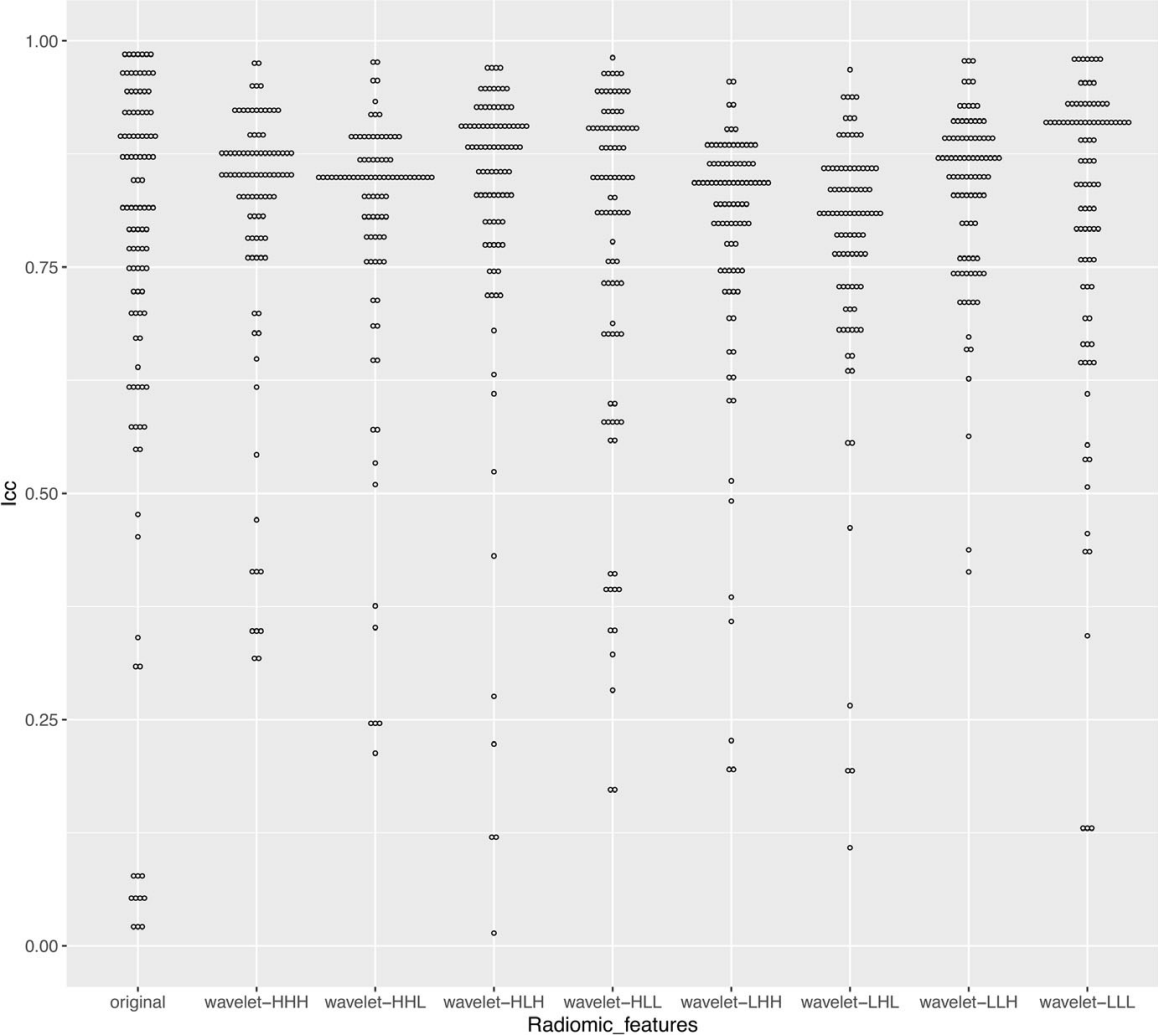
Wilkinson’s ICC (intraclass correlation coefficient) for radiomic features. All radiomic features are divided into nine groups: the original group (including 107 features) and eight wavelet groups (93 features for each).

Through texture analysis of each patient's chest CT, 851 radiomic features were obtained, including 107 original features and 8 groups of wavelet features (each group contains 93 wavelet feature factors) obtained by decomposition of the original features (except 14 shape features). With ICC > 0.75 as the screening criterion, 638 stable radiomic features were obtained, including 569 wavelet features and 69 original features (Fig. 2).

### 3.3 Prediction model development and ROC analysis

Eighty‐eight patients were randomly selected by SPSS as the training cohort, and the validation cohort consisted of the remaining 30 patients.

#### 3.3.1 Clinical prediction model

**Fig. 3:**
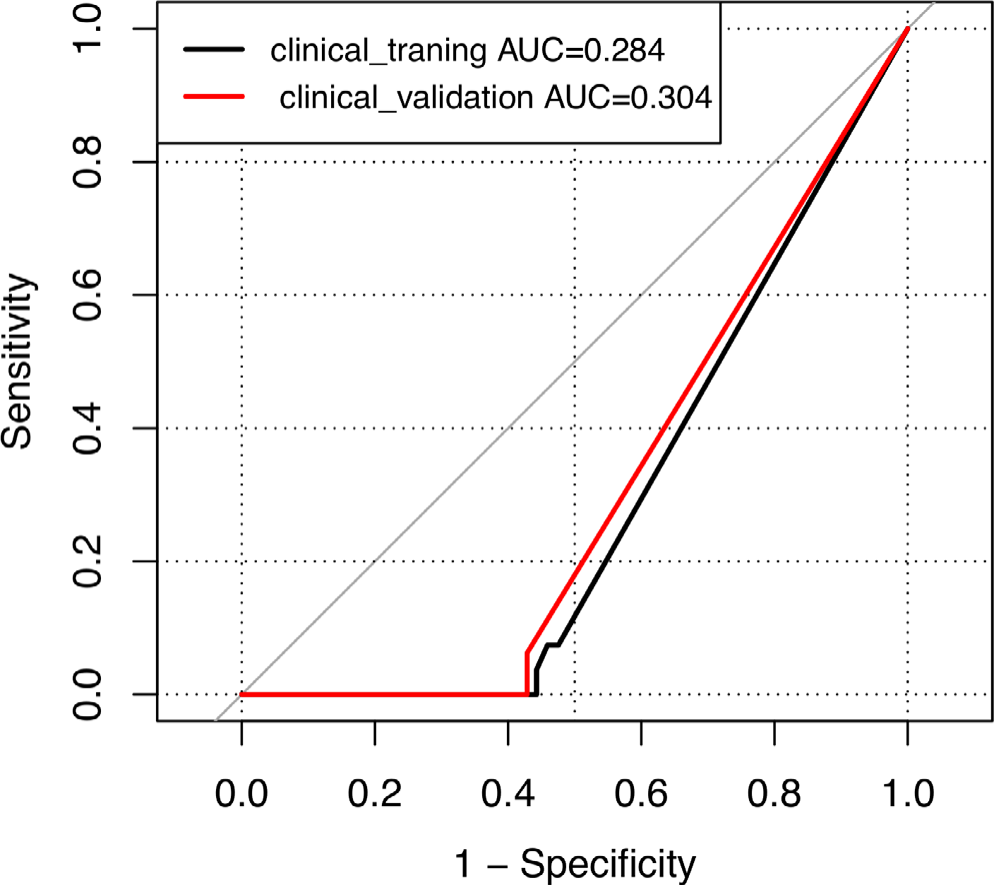
ROC curves for EGFR mutation prediction in the training group (clinical_training) and in the validation cohort (clinical_validation).

The logistic regression analysis results revealed that smoking index (*P* = 0.028) was a predictor of EGFR mutation in the training cohort of 88 patients. The ROC curve based on this plot was used to represent the clinical prediction model (clinical_training) of clinical features for EGFR mutation. As shown in Fig. 3, the smoking_index shown in the model was negatively correlated with EGFR mutation.

#### 3.3.2 General imaging prediction model

**Fig. 4:**
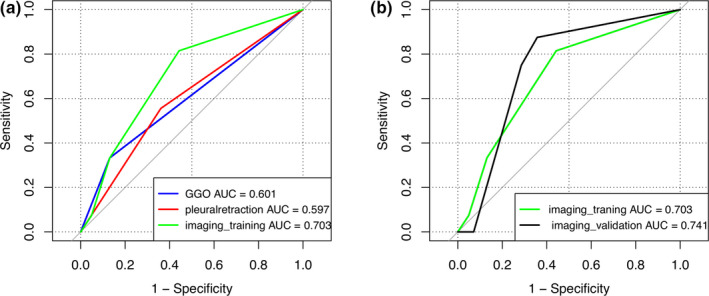
(a) ROC curves for EGFR mutation prediction with general imaging features separately and combined (imaging_combined). (b) ROC curves for EGFR mutation prediction in the training group (imaging_training) and in the validation cohort (imaging_validation).

In the training cohort of general imaging features, logistic regression analysis was performed, and the results revealed that GGO (p = 0.015) and pleural retraction (p = 0.041) were independent predictors of EGFR gene mutation. The ROC curve prediction model (imaging_training) based on general imaging features is shown in Fig. 4. The combination of the two models can significantly improve the predictive ability of EGFR mutation (imaging_training AUC = 0.703).

#### 3.3.3 Radiomic prediction model

**Fig. 5:**
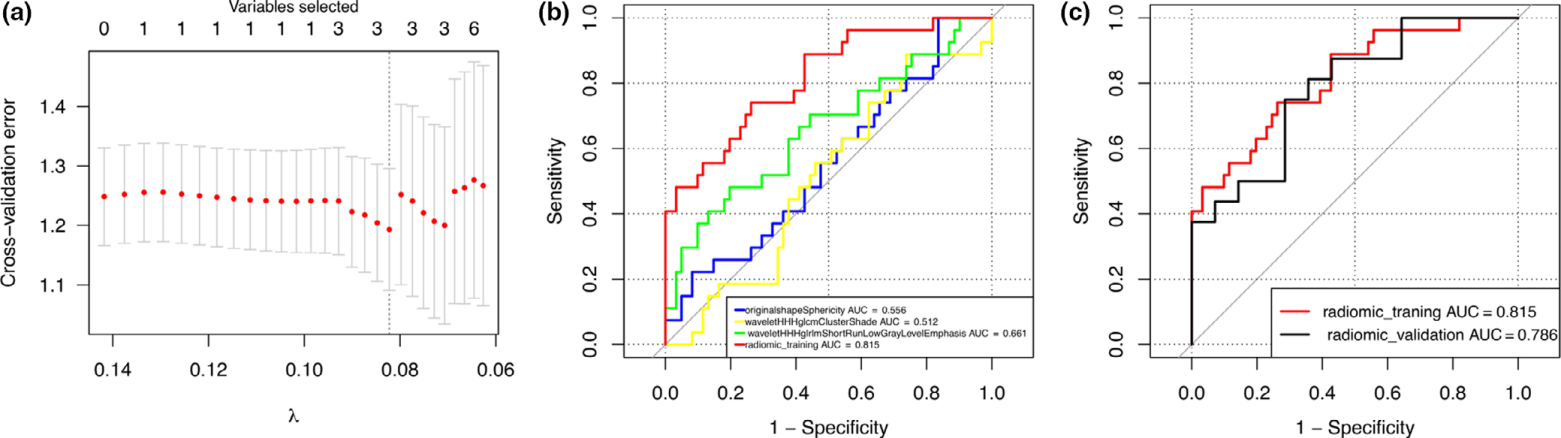
Radiomic feature selection and the development of the clinical prediction model. (a) The LASSO algorithm and 10‐fold cross‐validation for clinical predictor selection. The optimal lambda is 0.082 at the minimum cross‐validation error (1.19), and the corresponding number of predictors is 3. (b) ROC curve for EGFR mutation prediction with radiomic predictors separately and combined in the training cohort. (c) ROC curve for the training cohort (radiomic_training) and validation cohort (radiomic_validation), and the corresponding AUC was 0.815 and 0.786 (*P* = 0.762).

After MCP‐penalized LASSO regression analysis and tenfold cross‐validation of 638 radiomic features in the training cohort of 88 patients, the relationship between the cross‐validation error and the parameter lambda was determined and is depicted in Fig. 5. To avoid overfitting the model, the number of features was as few as possible. The optimal lambda is 0.082 at the minimum cross‐validation error (1.19), and the corresponding number of predictors is 3: wavelet_HHH_glrlm_ ShortRunLowGrayLevelEmphasis (*P* < 0.001), wavelet_HHH_glcm_ClusterShade (*P* = 0.031) and original_shape_Sphericity (*P* = 0.001). ROC curves were drawn based on these radiomic features. In the prediction model, the AUC of each texture feature ranged from 0.512 to 0.661. The predictive ability of a single texture feature for EGFR mutation was poor. The combined predictive ability of all texture features, radiomic_training, was 0.815, indicating improved predictive ability (Fig. 5).

#### 3.3.4 Combined prediction model

**Fig. 6:**
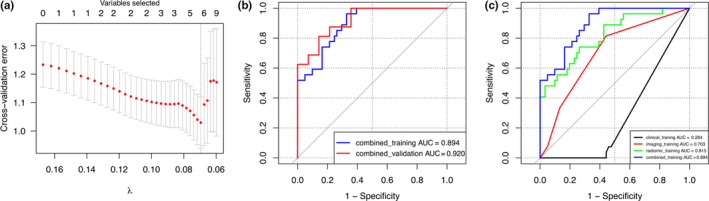
The development of the combined prediction model. (a) The LASSO algorithm and tenfold cross‐validation for combined predictor selection. When the minimum cross‐validation error is 1.03, the optimal lambda value is 0.695, and the corresponding number of nonzero coefficients is 5. (b) ROC curves of the combined prediction model for the training cohort (combined_training AUC = 0.894) and the validation cohort (combined_validation AUC = 0.920). (c) ROC curves are depicted to describe the discrimination of the clinical prediction model (clinical_training), the general imaging prediction model (imaging_training), the radiomic prediction model (radiomic_training) and the combined prediction model (combined_training).

Finally, all 647 factors (including 4 clinical features, 5 general imaging features, and 638 radiomic features) were analyzed by LASSO regression and tenfold cross‐validation to obtain the significant predictors for building the combined prediction model. As shown in Fig. 6, when the minimum cross‐validation error is 1.03, the optimal lambda value is 0.695, and the corresponding number of nonzero coefficients is 5: smoking_index, pleuralretraction, original_shape_Sphericity, wavelet_HHH_glcm_ClusterShade and wavelet_HHH_glrlm_ShortRunLowGray‐LevelEmphasis. The ROC curves in Fig. 6 show that the predictive ability of the combined prediction model was better than that of any single prediction model developed by clinical features, general imaging features or radiomic features.

The AUC, 95% CI, and the formula for calculating the score of the prediction models are shown in Table 3. No significant difference in AUC values was found between the training cohort and the validation cohort for any of the four prediction models.

**Table 3:** Features of the prediction models

Prediction models	Cohort	AUC	95% CI	*p*‐value*	Formula for calculating the model score	Value range of the models	
						EGFR+	EGFR‐
Clinical model	training	0.284	0.21‐0.357	0.815	Clinical‐score=‐0.225‐0.006∗A	−0.375 to −0.225	−1.665 to −0.225
	validation	0.304	0.156‐0.45				
Imaging model	training	0.703	0.594‐0.812	0.731	Imaging‐score=‐1.607+1.028∗B+1.437∗C	−1.607 to 0.858	−1.607 to 0.858
	validation	0.741	0.555‐0.927				
Radiomic model	training	0.815	0.718‐0.913	0.762	Radiomic‐score=2.309‐9.413∗D‐0.422∗E+8.165∗F	−2.605 to 4.488	−4.715 to 0.558
	validation	0.786	0.621‐0.95				
Combined model	training	0.894	0.829‐0.959	0.653	Combined‐score=1.35‐7.088∗D‐0.456∗E+7.844∗F+1.011∗C‐0.005∗A	−1.87 to 5.651	−14.145 to 0.854
	validation	0.920	0.828‐1				

A = smoking_index; B = GGO; C = pleural retraction; D = original_shape_Sphericity; D = wavelet_HHH_glcm_ClusterShade; E = wavelet_HHH_glrlm_ShortRunLowGrayLevelEmphasis.

* The *P*‐value was based on a comparison of AUCs between the training cohort and the validation cohort.

### 3.4 Establishment and validation of the nomogram

**Fig. 7:**
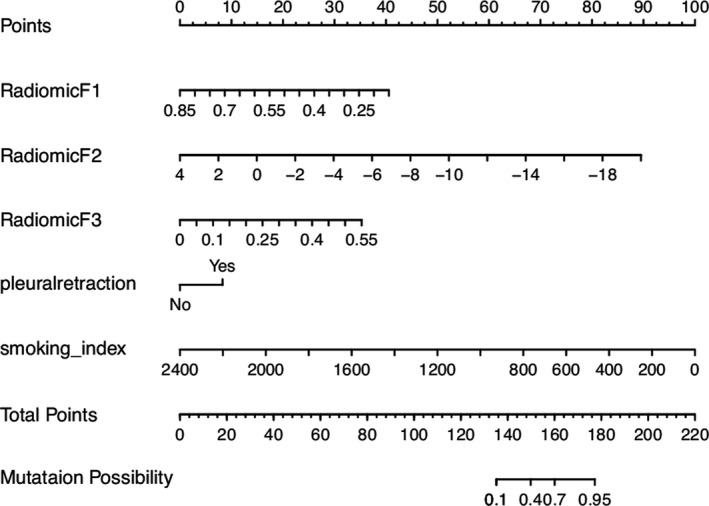
The nomogram that incorporated all the significant predictors for EGFR mutation was constructed with the training cohort. The predictors include RadiomicF1 (originalshapeSphericity), RadiomicF2 (waveletHHHglcmClusterShade), RadiomicF3 (wavelet‐HHHglrlmShort RunLowGrayLevelEmphasis), pleuralretraction and smoking_index.

**Fig. 8:**
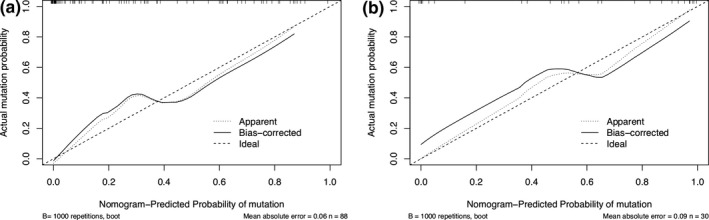
The calibration curve of the nomogram for predicting the probability of EGFR mutation in the training cohort (a) and the validation cohort (b). The actual mutation probability is plotted on the y‐axis; the nomogram‐predicted mutation probability is plotted on the x‐axis.

Based on the five predictors selected in the combined model, a nomogram was constructed to predict individual EGFR mutations. As shown in Fig. 7, the sum of points received for each variable value was located on the total points axis, and a line was drawn downward to the prediction axis to determine the mutation probability. The C‐index of the nomogram for mutation prediction was 0.894 (95% CI, 0.861 to 0.926) in the training cohort and 0.92 (95% CI, 0.875 to 0.965) in the validation cohort. The nomogram was subjected to 1,000 bootstrap resamples for internal validation, and the calibration curve was plotted (Fig. 8). The mean absolute error of calibration curves was 0.06 in the training cohort and 0.09 in the validation cohort.

## 4 DISCUSSION

The aim of this study is to establish a noninvasive predictive model of EGFR mutation based on clinical, imaging, and radiomic features, which can provide a basis for targeted therapy with patients who cannot be pathologically diagnosed with NSCLC and are unable to undergo EGFR gene mutation detection for various reasons. Therefore, the pathological types and tumor stages of the patients were not included in the analyses performed in this study.

Among the four clinical features included in the analysis, gender and smoking index were significantly different between patients with EGFR (+) and EGFR (‐) mutation status, but only smoking index was an independent predictor of negative EGFR mutation status. The AUC of the smoking index was 0.284 in the prediction model of EGFR mutation in the training cohort and 0.304 in the validation cohort. Previous studies showed that EGFR gene mutation occurred mostly in nonsmokers.^13^, ^15^, ^24^, ^25^, ^26^ A recent meta‐analysis based on 13 studies also suggested that smoking inhibited EGFR mutation in NSCLC (OR 0.28, 95% CI 0.21‐0.36, *P* < 0.01).^27^ Most studies have suggested that EGFR gene mutations were predominant in Asian nonsmoking women with adenocarcinoma, but gender was not an independent predictor of EGFR gene mutation in this study. This result may be related to the small sample size of this study.

Regarding the general imaging features, our study found that GGO and pleural retraction were independent predictors of a positive EGFR mutation status. Previous studies have suggested that GGO is a risk factor for EGFR mutation.^28^, ^29^, ^30^ Recent studies by Wang et al^31^ found that GGO volume percentages were significantly higher in patients with primary lung adenocarcinomas and EGFR mutation than in adenocarcinomas without EGFR mutation. This result could be related to the fact that EGFR mutation is significantly more common in lepidic predominant adenocarcinomas, which usually present as GGO‐predominant nodules on CT.^10^, ^32^ The results of these studies are consistent with those of our study. Nevertheless, some studies have drawn different conclusions. One study suggested that EGFR mutation status similar between GGO and solid adenocarcinoma, and the volume and diameter of GGO were related to EGFR mutation.^30^ Studies in 2011^33^ and in 2010^34^ found no significant correlation between EGFR mutation and GGO (*P* = 0.07 and *P* = 0.44). Zhang et al^27^ concluded that pleural retraction was a significant risk factor for EGFR mutation in NSCLC (OR 1.59, 95% CI 1.31‐1.92, *P* < 0.01) through a meta‐analysis of 11 studies including 2321 patients before August 2018. A recent study confirmed pleural retraction as an independent predictor of EGFR mutation again by multivariate regression analysis.^35^ In our study, the AUCs of GGO and pleural traction from ROC curves was 0.601 and 0.597, respectively, in the prediction model of EGFR mutation established by general imaging features. The combined predictive ability of GGO and pleural retraction was found to be improved (AUC = 0.703).

Texture analysis (TA) is an important means of medical image processing. In recent years, some studies have begun to apply TA to the evaluation of NSCLC gene mutations. However, the results of each study are not the same. Liu et al^36^ reported that EGFR mutation could be predicted by five radiological features that were divided into three groups: CT attenuation energy, tumor main direction, and texture defined by wavelets and laws (AUC 0.647). Another small sample study (25 EGFR mutations and 20 wild‐type EGFRs) found that contrast, correlation, and inverse difference moment radiomic features were associated with EGFR mutation status in lung adenocarcinoma.^37^ In a study of 298 patients, a radiomic GLSZM feature termed Size Zone NonUniformity Normalized (OR: 0.010, 95% CI: 0.0001‐0.852, *P* = 0.042) was found to be a risk factor for EGFR mutation.^19^ A multicentre study conducted in 2017^20^ found that 16 radiomic features were significantly correlated with EGFR mutation. In our study, original_shape_Sphericity, wavelet_HHH_glcm_ClusterShade and wavelet_HHH_glrlm_ShortRunLowGrayLevelEmphasis were the three radiomic predictors of EGFR mutation. Original_shape_Sphericity is a measure of the roundness of the shape of the tumor region relative to a sphere. A given volume in a sphere with the smallest possible surface area may have a higher probability of EGFR mutation. Wavelet_HHH_glcm_ClusterShade and wavelet_HHH_glrlm_ShortRun‐ LowGrayLevelEmphasis resulted from directional filtering of glcm_ClusterShade and glrlm_ShortRunLowGray‐LevelEmphasis with a high‐pass filter along the x‐direction, a high‐pass filter along the y‐direction, and a high‐pass filter along the z‐direction. Wavelet_HHH_glcm_ClusterShade is a measure of the asymmetry about the mean gray‐level intensity in the VOI and a higher value indicating the greater intratumor heterogeneity. Wavelet_HHH_glrlm_ShortRunLowGrayLevelEmphasis measures the joint distribution of shorter run lengths with lower gray‐level values and a greater value indicating more fine structural textures and more concentration of low gray‐level values in the VOI. Unfortunately, none of the above studies, including our own, have reported a common factor or model of radiomic features to predict EGFR mutation, which could be explained as follows: First, it could be due to the source of CT data; there is no standard requirement of DICOM raw data for CT texture analysis at present, and different CT machines and different CT scanning parameters could lead to different results from radiomic feature extraction. Second, different texture analysis software programs are used by different research institutes, which also contributes to the lack of consistency and repeatability in the final results. 3D Slicer is an open‐source software platform for medical image processing. In our study, we used the free software package Radiomic to extract radiomic features. We hope that this software is also used in similar research in the future to obtain more comparable results.

In the prediction model for EGFR mutation established by radiomic features, the predictive ability of a single feature is not strong, but the comprehensive predictive ability is significantly improved (AUC = 0.815). The combined prediction model, which combines the three groups of features, is much better than any single prediction model (AUC = 0.894). Limited by the predictive ability of a single prediction model, most of the related studies in the literature have used a combination of clinical features and general image features^35^, ^36^, ^38^ or a combination of clinical features and texture features^18^, ^19^, ^20^ to improve the predictive ability of the EGFR mutation prediction model. Only one study^21^ combined clinical features, general image features and radiomic features to establish a prediction model (AUC = 0.863) for EGFR mutation; however, only 11 original radiomic features were included in that study, and many wavelet transform features were excluded. We believe that in future research, the incorporation of noninvasive features such as pathological features and tumor marker features into the comprehensive prediction model may be more helpful for improving the predictive ability for EGFR mutation.

The nomogram established by smoking_index, pleuralretraction and three radiomic features performed well in predicting EGFR mutation. It is an intuitive individual prediction model, and its prediction ability is supported by the C‐index (0.894 and 0.92 for the training and validation cohorts, respectively) and the calibration curve.

Limited by the small sample size, patients with EGFR exon 18, 19, 20, and 21 mutations were not analyzed separately in the present study. We hope that a large cohort of patients can be enrolled in future studies for further analysis.

## 5 CONCLUSIONS

Smoking index, pleural retraction, and three radiomic features were identified as independent prognostic factors of EGFR mutation status in NSCLC. Radiomic features are better predictors than general imaging features or clinical features. Our study may help develop a noninvasive biomarker to identify EGFR mutation status by using a combination of the three group features.

## AUTHORS' CONTRIBUTIONS

Dang YT was responsible for project conceptualization, data analysis, writing of the manuscript, and all manuscript revisions. Wang RT and Qian K were responsible for patient data collection. Lu J was responsible for CT data collection. Zhang HX was responsible for statistical analysis. Zhang Y was responsible for project conceptualization, manuscript revisions, and editing of the manuscript. All authors read and approved the final manuscript.

## CONFLICT OF INTERESTS

No conflict of interest exists.

## ETHICS APPROVAL

All procedures performed in studies involving human participants were in accordance with the ethical standards of both institutional and national research committees and with the 1964 Declaration of Helsinki and its later amendments or comparable ethical standards.

## CONSENT TO PARTICIPATE

Informed consent was obtained from all individual participants included in the study.

## CONSENT FOR PUBLICATION

Not applicable.

## CODE AVAILABILITY

All codes used with R are available in the Appendix.

## Supporting information


**Appendix**: Related computerized programs for statistical analysis with R.

## Data Availability

The datasets used and analyzed during the current study are available from the corresponding author upon reasonable request.
