# Supplementary material for: Clinical and radiological predictors of epidermal growth factor receptor mutation in nonsmall cell lung cancer
Source: J Appl Clin Med Phys. 2020 Dec 12;22(1):271–80. doi: 10.1002/acm2.13107 (PMC7856515; doi:10.1002/acm2.13107)
Supplement: Supplementary file 1 — Appendix: Related computerized programs for statistical analysis with R. [file ACM2-22-271-s001.docx]

**Related Computerized Programs for Statistical Analysis With R**

**For intraclass correlation coefficient (ICC) Calculation**

T12 <- cbind(T1,T2);dim(T12)

library(psych)

t= 2

icc <- c(1:y)

for(i in 1:y) {icc[i] <- ICC(T12[,c(i,i+y)])$results$ICC[t]}

**For logistic regression**

library("MASS")

glm <- glm(Y ~ age…, family = binomial(link = logit), data =df)

logit.step <- step(glm, direction = "backward")

**For LASSO regression and 10-fold cross-validation**

library(ncvreg)

X <- as.matrix(df[,1:638])

y <- df$mutationstatus

n <- dim(X)[1]

p <- dim(X)[2]

cv.out <- cv.ncvreg(X,y,family="binomial",penalty="MCP",nfolds=10)

plot(cv.out,log.l=FALSE)

**For ROC**

library(pROC)

library(ggplot2)

roc1 <- roc(df$mutationstatus, df$originalshapeSphericity)

roc2 <- roc(df$mutationstatus, df$waveletHHHglcmClusterShade)

roc3 <- roc(df$mutationstatus, df$waveletHHHglrlmShortRunLowGrayLevelEmphasis)

roc4 <- roc(df$mutationstatus, df$PRE)

plot(roc1, legacy.axes=TRUE, add=T,col="blue", grid=c(0.5, 0.2),grid.col=c("black", "black"))

plot(roc2, legacy.axes=TRUE, add=T,col="yellow")

plot(roc3, legacy.axes=TRUE, add=T, col="green")

plot(roc4, legacy.axes=TRUE, add=T, col="red")

legend=legend("bottomright", legend = c("originalshapeSphericity AUC=0.556", "waveletHHHglcmClusterShade AUC=0.512"," waveletHHHglrlmShortRunLowGrayLevelEmphasis AUC=0.661","radiomic_training AUC=0.815"),col = c("blue","yellow","green","red"),cex = c(0.5), seg.len=1, text.width=0.87, x.intersp=0.1,y.intersp=0.8, xpd = "TURE", lwd =2)

**For nomogram**

library("rms")

mod=lrm(f,training,x=T,y=T)

nom=nomogram(mod,fun=plogis,fun.at=c(0.1,seq(0.1,0.9,by=0.3),0.95),

lp=F,funlabel="Mutataion Possibility")

plot(nom)

**For Computing the C-Index and 95% CI**

Library("Hmisc")

Cindex=rcorrcens(training$mutationstatus ~ predict(mod), data = training)

95%CI, 1.96 × se; se = S.D./2

**For Calibration Curve**

cal=calibrate(mod, cmethod='KM', method="boot",B=1000)

par(mar=c(8,5,3,2),cex = 1.0)

plot(cal,lwd=2,lty=1,errbar.col=c(rgb(0,0,255,maxColorValue=255)),xlim=c(.0,1.0),ylim=c(.0,1.0),xlab="Nomogram-Predicted Probability of mutation",ylab="Actual mutation probability",col=c(rgb(255,0,0, maxColorValue=255)))
